# The Effect of Blindness on Spatial Asymmetries

**DOI:** 10.3390/brainsci10100662

**Published:** 2020-09-23

**Authors:** Luca Rinaldi, Andrea Ciricugno, Lotfi B. Merabet, Tomaso Vecchi, Zaira Cattaneo

**Affiliations:** 1 Department of Brain and Behavioural Science, University of Pavia, Piazza Botta 6, 27100 Pavia, Italy; vecchi@unipv.it; 2 IRCCS Mondino Foundation, 27100 Pavia, Italy; andrea.ciricugno@mondino.it (A.C.); zaira.cattaneo@unimib.it (Z.C.); 3 The Laboratory for Visual Neuroplasticity, Department of Ophthalmology, Massachusetts Eye and Ear Infirmary, Harvard Medical School, Boston, MA 02115, USA; lotfi_merabet@meei.harvard.edu; 4 Department of Psychology, University of Milano-Bicocca, 20126 Milano, Italy

**Keywords:** visual deprivation, hemispheric asymmetry, laterality, spatial asymmetries, blindness

## Abstract

The human cerebral cortex is asymmetrically organized with hemispheric lateralization pervading nearly all neural systems of the brain. Whether the lack of normal visual development affects hemispheric specialization subserving the deployment of visuospatial attention asymmetries is controversial. In principle, indeed, the lack of early visual experience may affect the lateralization of spatial functions, and the blind may rely on a different sensory input compared to the sighted. In this review article, we thus present a current state-of-the-art synthesis of empirical evidence concerning the effects of visual deprivation on the lateralization of various spatial processes (i.e., including line bisection, mirror symmetry, and localization tasks). Overall, the evidence reviewed indicates that spatial processes are supported by a right hemispheric network in the blind, hence, analogously to the sighted. Such a right-hemisphere dominance, however, seems more accentuated in the blind as compared to the sighted as indexed by the greater leftward bias shown in different spatial tasks. This is possibly the result of the more pronounced involvement of the right parietal cortex during spatial tasks in blind individuals compared to the sighted, as well as of the additional recruitment of the right occipital cortex, which would reflect the cross-modal plastic phenomena that largely characterize the blind brain.

## 1. Introduction

At first glance, the brain appears to be a symmetrical structure; however, a closer inspection reveals lateralized changes from the subcellular and neurochemical to gross anatomical levels [1]. These laterality patterns are not limited to the level of the brain but pervade as well in traits of human overt behavior such as handedness (the most frequently observed and studied behavioral asymmetry [2]) and spatial asymmetries as measured by line bisection tasks. Lateralization phenomena in the context of the line bisection task have been ascribed to genetic variation with dopaminergic system genes [3,4] or the allelic variation in genes affecting corpus callosum structure [5] associated with the direction (left versus right) and the magnitude of spatial orienting bias. However, investigating the role of environmental factors [6], recent research has suggested that epigenetic regulation contributes to the development of hemispheric asymmetries subserving spatial processing in line bisection tasks [7]. This may indicate that the bias in the line bisection task is likely influenced by multiple genetic, epigenetic, and environmental factors.

Within this theoretical framework, the study of visual deprivation represents a unique model to investigate lateralization phenomena, as experiential factors are clearly different compared to typical development. For instance, whereas blind and sighted individuals are exposed to language (a typical left-lateralized function in the brain [8]) to similar degrees, their spatial abilities (typically recruiting more the right hemisphere [9]) develop based on haptic and auditory input. In principle, the lack of early visual experience may thus affect the lateralization of spatial functions, which in the blind rely on a different sensory input compared to the sighted. Compensatory phenomena occurring at the brain and behavioral levels following visual deprivation have been extensively studied (for reviews see References [10,11,12]). However, how blindness affects the level of hemispheric asymmetry and laterality patterns in spatial processing is an issue that has not reached definite conclusions.

This review focuses on available evidence associated with lateralization patterns in spatial processes in the blind. Spatial functions are characterized in sighted individuals by specific lateralization patterns at the neural level that, at times, are reflected by asymmetric behavioral profiles. Here, we review evidence on behavioral and brain asymmetries consistently observed in the blind in spatial processing both at the perceptual level (i.e., auditory and tactile) and at the attentional and representational level (i.e., orienting spatial attention in physical or mental space as in case of the mental number line). In particular, we begin by reviewing the effects of visual experience on spatial asymmetries in bisection tasks and then discuss the available evidence on spatial laterality patterns as gathered from mirror symmetry and localization tasks (Table 1 reports the key studies reviewed and their relative sample sizes). In our review, in analogy with the classification adopted by most previous works, congenital (i.e., usually defined as those individuals blind from birth) and early (i.e., usually defined as those individuals who become blind during early postnatal life, generally around 2 or 3 years of age) blind participants will be treated as a single group.

## 2. Pseudoneglect

An overwhelming body of evidence suggests that spatial attention functions are largely lateralized to the right hemisphere (e.g., [25,26]). One of the most prominent manifestations of such hemispheric asymmetry comes from the investigation of lateralized behavior in line bisection tasks, where participants are asked to manually estimate the midpoint of a horizontal visual line. In these tasks, neurotypical individuals exhibit a slight but consistent leftward bisection error; a bias known as *pseudoneglect* (for a review see Reference [27]), which is thought to depend on the dominant role played by the right hemisphere in orienting spatial attention resources [28,29,30,31]. Interestingly, although being susceptible to different types of experimental manipulations (such as line length, cueing, and the location of space where the lines appear), pseudoneglect has been shown to occur in both the visual and haptic modalities [32,33].

**Figure 1:**
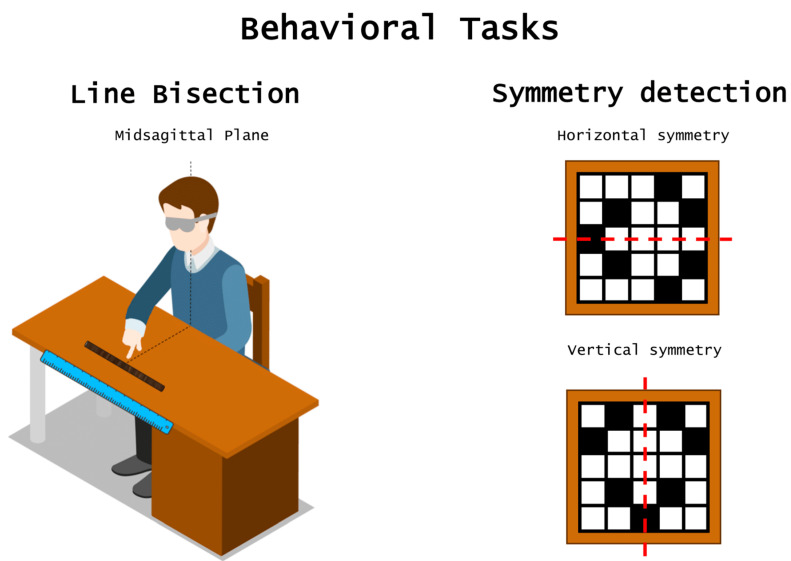
Behavioral tasks used to assess spatial asymmetries. A typical task probing the presence of spatial asymmetries in spatial processing is the haptic line bisection task in which participants are asked to indicate the midpoint of a horizontal rod. Various studies indicate that blind individuals generally show a leftward bias when bisecting the horizontal rod as sighted individuals do. Symmetry detection may be also informative about laterality patterns following visual deprivation. In this case, participants are asked to haptically explore a 2D matrix and must remember the position of target cells, which may be arranged in a symmetrical (i.e., either on the horizontal or vertical axis) or non-symmetrical configuration. Whereas sighted individuals show a clear advantage for symmetry detection along the vertical axis as compared to the horizontal one, no such a difference is observed in early blind individuals.

A critical question is whether such a leftward bias is driven by normal visual development and experience. Available findings suggest that this is not the case, since early blind individuals also show pseudoneglect in the way they represent space. For instance, in an early study by Bradshaw and colleagues [13], participants had to adjust the extremities of a rod protruding from a copper tube. These investigators found that most of the early blind adults overestimated the left side of space, resembling the pattern of performance displayed by sighted individuals. These findings were replicated more recently in a haptic line bisection paradigm, where participants were asked to estimate the midpoint of a horizontal rod with a leftward bias consistently reported in blind individuals (Figure 1) [14,15]. Interestingly, the magnitude of pseudoneglect was even larger in blind than in blindfolded sighted participants [14,15]. The authors go on further to suggest that this observed bias may depend on the intense spatial training experienced by blind individuals (e.g., high level of independent mobility) that may have contributed to this lateralization of function in the control of spatial attention (see also Reference [34]).

With the aim of understanding whether and how the lack of visual input from only one eye affects the typical pattern of hemispheric asymmetry in the control of spatial attention, a recent study tested twelve monocular blind individuals (eight left-eye blind with functioning right eye, and four right-eye blind with functioning left eye) with a visual and a haptic line bisection task [35]. Results showed that when participants were asked to visually bisect lines, monocular blind subjects displayed an overall tendency to bisect toward the direction of the functioning eye, possibly reflecting a preferential activation of the contralateral hemisphere of the functioning eye [36]. However, this pattern did not reach statistical significance (note that the sample size was quite small, thus limiting the statistical power of the study). Interestingly though, monocular blind participants showed a consistent leftward bisection bias in the haptic modality (i.e., with no visual input available), in line with prior studies with normally sighted and blind participants [15]. These data indicate that the effects on spatial attention related to monocular blindness may only pertain to the visual modality.

Taken together, these findings suggest that the right-hemispheric dominance for spatial processing develops even in the absence of early visual experience. Interestingly, pseudoneglect was also reported in early blind children aged 7–11, despite the fact that in this study, sighted children did not display a leftward bias [16]. In the study by Sampaio and colleagues [16], the experimenter placed the child’s index finger at one of the two ends of the rod and asked them to run their finger along the wooden rod as many times as they wanted and then to stop at the place they estimated to be the midpoint. The presence of a leftward bias in blind children reported in this study [16] may suggest that the lack of visual experience can even anticipate or strengthen hemispheric lateralization for spatial processing during development. Yet, such findings should be corroborated by further empirical works, especially because pesudoneglect has been reported in more recent studies involving children of this age. These studies indeed indicate that pseudoneglect emerges gradually over developmental time, as indexed by both the visual line bisection [37] and cancellation [38] tasks. Such a gradual shift as a function of age has been interpreted as a sign of literacy acquisition (i.e., reading and writing habits, which are oriented from left-to-right in Western countries; [38]). New studies are thus needed to substantiate the possibility that blind children show a more pronounced leftward bias in the haptic bisection task.

At the neural level, visuospatial attention orienting (as measured by line bisection task performance) in sighted individuals is mediated by a right fronto-parietal network, with a key role played by the posterior parietal cortex [31,39,40,41]. Although no study has directly measured brain responses in blind participants whilst performing a bisection task, it is likely that the blind would also show a preferential activation in their right fronto-parietal network. Indeed, a right fronto-parietal activation has been reported in the blind in tasks implicated with spatial processing, such as spatial imagery (e.g., angle discrimination), auditory and haptic localization [42,43,44,45,46].

Biases in spatial attention do not only pertain to the horizontal space, but also to the vertical and radial planes. Neurologically healthy participants typically err away from their body in radial bisection tasks [47,48,49], and in the upward direction along the vertical axis [50]. Although the role of hemispheric asymmetry in the control of spatial attention in the vertical and radial planes is not fully established [30,51], it is worth mentioning that early blind participants did not show consistent spatial biases in haptic radial and vertical bisection tasks, except for biases driven by the final movement direction [15].

Behavioral asymmetries in the sighted, similar to the perceptual ones observed for the line bisection task in both visual and tactile domains, have been reported as well for visuospatial representations held in long-term memory [52,53,54,55]. This fascinating phenomenon, known as *representational pseudoneglect* [55], has been documented for both remembered spatial information (i.e., not perceptually available) and purely abstract information such as numbers. For instance, adult individuals (in Western cultures that read left-to-right) typically represent numerical information along a mental number line, with smaller numbers associated with the left side of the space and larger numbers with the right side of the space [56]. The task typically used to explore whether representational pseudoneglect also occurs for the mental number line is the bisection of numerical intervals [57,58]. In this task, participants are presented with two numbers and are asked to identify the midpoint of the numerical interval (i.e., a “gut” response is required, without explicit calculation). In analogy with pseudoneglect for physical lines, neurotypical individuals generally display a leftward bias, thus erring in the direction of the lower number in the pair [57,58,59].

Blind individuals seem to represent numbers as sighted individuals do; that is, in the form of a left-to-right oriented mental number line [19,60,61], a representation that is likely to engage a similar parietal network in both the blind and the sighted as shown by electrophysiological evidence [62]. Critically, blind individuals display a consistent leftward bias when performing an auditory numerical bisection task, with the magnitude of this bias being comparable to that shown in sighted controls [61]. In this task, participants are typically presented aurally with pairs of numbers (e.g., “117–166”) and have to judge and orally report the numerical midpoint of the number pair immediately after the presentation of each auditory stimulus without explicit calculation (i.e., a time limit is generally imposed in order to prevent participants from calculating the middle value). The fact that both groups display a similar leftward bias in numerical bisection tasks supports the view of a hemispheric asymmetry in the control of spatial attention in blind individuals that extends to mental representations (i.e., beyond physical space). Accordingly, the mental representation of numbers has been found to interact with the representation of physical space in the blind as it does in sighted participants. For instance, in both sighted and blind participants, listening to small numbers during haptic line bisection increases pseudoneglect [18]; furthermore, tapping hand movements in either the left or right peripersonal space while bisecting numerical intervals modulates the leftward bisection numerical bias to a similar extent in both right-handed blind and sighted individuals, with this effect also depending on the hand used to tap [19].

Although the mechanisms responsible for the orientation of attention in external space are likely to be distinct from those operating along the mental number line [63], the experimental evidence reviewed above suggests that the right-hemispheric dominance in processing of both physical and representational (abstract) space develops even in the absence of any visual input.

## 3. Mirror Symmetry

Insights on lateralization patterns in spatial processing following visual loss may also come from recent studies that focused on mirror symmetry detection. Symmetry is ubiquitous, characterizing both natural organisms and human constructions including art. Accordingly, mirror symmetry is detected effortlessly and very rapidly (i.e., in a few tens of milliseconds) by the human visual system, especially when symmetry is along the vertical plane, which is considered the most salient axis also because of the lateral position of the eyes (for a review see Reference [64]). Interestingly, symmetry acts as a grouping principle of perceptual organization, even when it is haptically perceived (i.e., without any available visual input), both in sighted and blind individuals [65]. However, the vertical axis of symmetry does not seem to be more salient than other axes in early blind individuals, a finding suggesting that the vertical axis salience may depend on normal exposure to vertical symmetry in the visual world [21,22,66]. For instance, in a study by Cattaneo and colleagues [21] blind and sighted participants were presented with a short-term memory task in which they had to memorize and retrieve a series of target cells on a 2D matrix that had to be explored in the haptic modality. Crucially, target cells could either form a symmetric spatial configuration (i.e., along the vertical or the horizontal axis) or be arranged in a random, non-symmetrical layout (see Figure 1). The results showed that while sighted participants exhibited a better memory recall for the vertical configurations as compared to the horizontal ones, no such difference characterized the performance of early blind individuals thus suggesting that visual experience does play a crucial role in symmetry detection [21].

At the neural level, early functional magnetic resonance imaging (fMRI) studies in sighted individuals have shown that extrastriate visual regions including the lateral occipital (LO) cortex are bilaterally activated during visual symmetry detection [67,68]. Yet, more recent studies employing non-invasive brain stimulation have indicated that symmetry detection may recruit more areas in the right hemisphere; in particular, the right LO cortex, further extending to the right occipital face area (OFA), a key area in face processing [69,70,71]. Indeed, one of the factors contributing to such a hemispheric asymmetry for symmetry detection may be represented by the right-hemispheric dominance in face processing (with symmetry being a critical cue in face detection [72]). The study of visual deprivation represents an interesting opportunity to corroborate this hypothesis. In fact, if hemispheric asymmetries in mirror symmetry detection are mainly driven by the right-lateralized network for face processing; we may expect no such a lateralized pattern in the early blind given that they do not have extensive visual experience with faces.

The only fMRI evidence available regarding the neural correlates associated with symmetry detection in the blind is the study by Bauer et al. [20]. Bauer et al. [20] required blind and sighted individuals to detect tactile symmetrical patterns. No specific lateralization pattern emerged in the LO cortex in either the blind or sighted participants; this is in agreement with prior neuroimaging evidence in the sighted [67,68]. Yet, to gather causal evidence from the putative role of visual experience in shaping hemispheric asymmetries in symmetry perception, future studies should explore the effect of transient disruption of right versus left LO cortex activity in blind individuals through non-invasive brain stimulation.

## 4. Localization Tasks

Consistent evidence from testing sighted individuals suggests that spatial processing and navigation in the visual world recruits the right more than the left hemisphere [73]. Such right-hemispheric dominance has also been reported in sound and tactile localization. Indeed, a large body of evidence suggests a right-hemispheric dominance for auditory spatial processing in humans with a crucial role of right parietal areas [74,75]. Similarly, tactile localization relies on a clearly asymmetric network, with the ability to localize objects in contact with the skin mediated by the right temporo-parietal junction [76]. This then begs the question: what is the role of a normal visual development in generating such hemispheric asymmetries? The lack of visual experience often leads to superior ability in sound and tactile localization [77,78], raising questions whether these functions are supported by more lateralized networks in the blind.

Blind individuals have been shown to have a three-dimensional spatial mapping of sound sources with equal or better precision than sighted individuals [78]. Further, whereas sighted and late blind individuals show severe impairments in localizing sounds monaurally [79], early blind individuals are able to localize a sound source under both monaural and binaural testing conditions [44,78,80]. Yet, despite evidence of enhanced abilities of blind individuals in detecting auditory stimuli originating from the periphery, this does not inform us thoroughly about any lateralization patterns following visual deprivation.

Direct indication about relative spatial biases in sound localization comes from a study by Vercillo and colleagues [24]. This study compared blind and sighted participants’ performance in a simple pointing task with static or moving sounds along the horizontal plane as well as in a task with moving sounds and head movements. Vercillo et al. [24] found that rotational head movements impaired sound localization in blind but not in sighted individuals with a bias in the direction of head motion in blind participants (suggesting a higher reliance in the blind on a body-centered reference frame). Critically, whereas no bias was observed in sighted subjects in the static condition, the group of early blind participants displayed a significant mislocalization toward the left. Such a dissociation resembles the pattern observed in the tactile bisection task in which a larger leftward bias has been consistently observed in blind subjects as compared to sighted controls [14,15].

In considering the leftward bias observed in blind (but not sighted) individuals for sound localization, it is worth mentioning neuroimaging studies that reported a specific recruitment of the right dorsal extrastriate occipital cortex during auditory spatial processing [46,81,82]. Accordingly, TMS delivered to the right occipital cortex in blind individuals affected sound localization abilities, especially for sounds originating from the left hemispace [23]. On the contrary, TMS delivered over the right intraparietal sulcus (rIPS) did not affect spatial processing of sounds in blind individuals, whereas it did so in sighted participants [23,83], suggesting that spatial localization abilities may rely also and possibly more on right-occipital than right-parietal regions in the blind. It is likely that the further recruitment of right occipital regions (beyond the typical fronto-parietal network) during spatial processing may contribute in the blind to strengthen a leftward bias in tasks tapping on spatial processing such as line bisection or sound localization.

## 5. The Role of Sensorimotor Experience

Visuospatial asymmetries in healthy individuals have long been interpreted primarily in terms of hemispheric activation [84,85]. According to the hemispheric activation hypothesis, the spatial nature of the task (e.g., line bisection task or cancellation task) would induce a preferential activation of the right hemisphere, leading in turn to an overestimation of the left hemispace and, thus, to the leftward bias known as pseudoneglect. This interpretation is a consequence of the Kinsbourne’s activation–orientation theory, which maintains that the attentional resources are located in the contralateral space of the most activated hemisphere [86,87]. Accordingly, various neuroimaging studies have reported a specific activation of the posterior parietal cortex during visuospatial tasks [39,40,41].

Despite the large consensus on a neurobiological basis of visuospatial asymmetry, recent evidence has pointed to the possible role of experiential factors (and, specifically, of sensorimotor experience) in modulating pseudoneglect. Over the past years, it has been repeatedly shown that cultural practices, such as reading habits, broadly influence spatial processes at both perceptual and representational levels. Critically, evidence for a cultural shaping of pseudoneglect-like biases is also not lacking. That is, reading habits have been found to influence line bisection tasks with readers of left-to-right oriented languages showing a leftward bias and readers of right-to-left oriented languages displaying an opposite, rightward bias [88,89,90]. These cross-cultural differences indicate that visuospatial asymmetries would reflect the tendency to scan information in the direction in which one reads. Because reading and writing routines permeate our everyday life across the entire lifespan, such directional sensorimotor practices would result in the allocation of more attentional resources in the hemispace in which one starts to read and write. This is also supported by developmental evidence indicating that the leftward bias in Western children emerges gradually over the school years with the formal introduction of reading and writing practices [37,38]. Accordingly, an interaction between biological and cultural (i.e., linked to sensorimotor experience) factors has been proposed to account for these findings with sensorimotor practices, such as reading and writing habits, that can either reinforce or modulate the leftward bias arising from the right hemisphere dominance in spatial processing [90].

The possibility that the lack of visual experience may strengthen the hemispheric lateralization for spatial processing (i.e., as indexed by the more pronounced leftward bias reported in the blind in bisection tasks) should be considered in the context of the several compensatory mechanisms that blind individuals need to develop in order to cope with their visual deficit. Tactile and auditory information are crucial for blind individuals to explore and represent the surrounding environment [10]. Hence, blindness imposes great demands on other sensory systems to make compensatory adjustments in the absence of sight. To account for the greater leftward bias showed by early blind individuals, we first pinpoint that as a consequence of neuroplastic reorganization, parietal cortical areas would be greatly implicated during tactile tasks (such as the line bisection task) and would be characterized by specific functional networks with strengthened occipitoparietal connectivity, as indicated by previous studies (e.g., [91]). Second, the greater demands on other sensory channels would be clearly reflected in the reading system blind individuals have to learn starting from early infancy, that is, Braille reading. In analogy with visual reading in Western countries, Braille reading proceeds from left-to-right. Yet, in contrast to visual reading, Braille is a tactile system in which the reader has to haptically scan specific patterns using the fingers. In fact, individuals who learn to read Braille must acquire the capacity to extract spatial information from subtle tactile stimuli [92]. As such, Braille reading imposes a marked increase in afferent and efferent demands of spatial computations, leading in turn to striking adaptive changes in the human brain [92]. Crucially, Braille reading has been shown to engage the occipital cortex, with recruitment being greater for more proficient Braille readers (i.e., greater occipital recruitment for earlier/longer visual deprivation compared to late blind participants) [93,94]. Such an experience-driven plasticity can be observed even in sighted adults who learn Braille after a relatively long training period (e.g., 9 months) with the activation of the occipitotemporal cortex modulated by the individual’s proficiency (i.e., as indexed by Braille reading speed) [95]. In Braille readers, the nature of the line bisection task (i.e., presented in the haptic modality) may therefore activate the right occipital cortex and contribute to the more pronounced leftward bias observed as compared to sighted individuals. Whether Braille practice is responsible for the asymmetrical hemispheric functions subserving the enhanced leftward bias in the blind is an open question that deserves future study, which should ideally compare the performance of Braille and non-Braille readers in spatial tasks. A similar argumentation can apply as well for the leftward bias reported only in blind (and not sighted) participants for sound localization.

Sensorimotor experience may also be crucial in the lateralization of spatial processes that subserve mirror symmetry detection. We have previously discussed the possible role of visual experience (especially with faces) in determining the vertical symmetry advantage over the horizontal one in sighted individuals. This possibility seems to be further supported by evidence from late blind individuals. Indeed, while no advantage has been observed in early blind individuals, late blind participants are significantly more accurate in remembering vertically compared to horizontally symmetric configurations when presented in the frontal plane, in analogy with sighted participants [22]. The higher salience of vertically symmetric patterns in the late blind for the frontal presentation of symmetrical configurations likely reflects a sort of visual “imprinting”. That is, previous experiences of visual vertical symmetry were reinforced by the congruent external frame during frontal presentation, resulting in the vertical symmetry advantage observed in late but not in early blind individuals.

## 6. Conclusions

**Figure 2:**
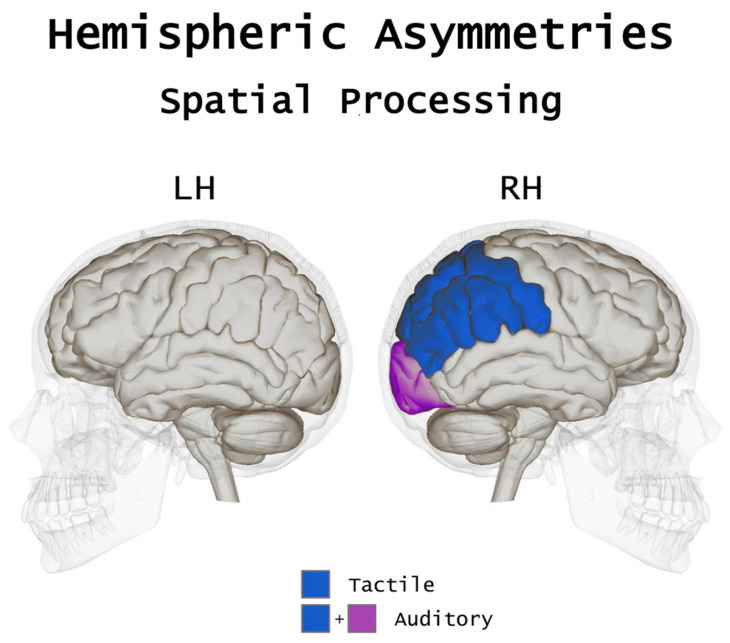
A pictorial, simplified view of the hemispheric asymmetries subserving spatial processing in the blind. In spatial tasks, in addition to a robust activation of parietal areas in tactile spatial processing, the blind also show a cross-modal recruitment of the occipital lobe, especially in auditory and tactile localization tasks.

Overall, the debate as to what extent visual experience contributes to a different development of functional asymmetries subserving spatial processing has not reached definite conclusions. Yet, available evidence indicates that the hemispheric dominance for spatial processing seems, overall, largely unaffected by visual loss, as in both sighted and blind individuals’ spatial functions would predominantly rely on the right hemisphere (see Figure 2). Beyond the common lateralization pattern, spatial processes in the blind seem to be supported by a more extended cortical network in the right hemisphere, which would be responsible for the more pronounced leftward bias in spatial tasks as compared to the sighted. Despite right parietal activations in both the blind and sighted brain have been consistently reported across a series of studies in spatial processing, parietal areas of the blind brain would elaborate a larger amount of information during tactile tasks (such as the line bisection task) as compared to the sighed (see Reference [91]). This, in addition to the cross-modal recruitment of areas in the occipital cortex, may contribute to determining the larger leftward bias reported.

Because spatial processing in the blind relies on a different sensory input compared to the sighted (i.e., where vision represents the primary sensory modality), the right hemisphere dominance for spatial functions in the blind may appear surprising. Yet, several brain areas have been shown to process information content independently of the sensory modality through which that information is conveyed. Such an ability of the human brain to process and represent specific information content in a more abstract manner, defined as supramodality, has been repeatedly reported in the blind (for a review, see Reference [12]). Interestingly, recent studies on blind individuals indicate that also the neural mechanisms subserving spatial perception and imagery are supramodal in nature (for a review, see Reference [12]). Hence, regardless of the specific modality through which information would be processed (i.e., whether visual, auditory or haptic), the right parietal cortex would play a crucial role in orienting spatial attention resources. Accordingly, the superior parietal and intraparietal cortices appear to play a crucial role in spatial representation in both sighted and early blind individuals and, thus, independently from visual experience [96]. Crucially, an influence of visual experience on the lateralized brain network subserving spatial processing would be observed in the form of cross-modal plastic phenomena [97,98]. Evidence from early blind individuals, indeed, indicates that the occipital cortex is cross-modally recruited in the processing of non-visual information, such as auditory, linguistic, and tactile stimuli [99,100]. In line with this evidence, the cross-modal recruitment of the right occipital cortex may contribute, alongside with the involvement of the right parietal cortex, in determining the greater behavioral lateralization (i.e., leftward bias) observed in different spatial tasks in early blind individuals.

Finally, it is worth considering that despite blindness profoundly affects how an individual interacts with the surrounding environment, there are many other experiential situations that can affect the heterogeneous origins and manifestation of lateralization patterns. For instance, none of the studies reviewed here has assessed possible additional variability due to the cross-cultural differences in blind individuals (for recent views on how culture and genes co-shape human behavior and brain see Reference [101]). This is particularly relevant, as many forms of spatial asymmetries have been shown to rely on cultural habits. Future research should thus aim at addressing the possible interactive effects of visual deprivation and cultural habits as well as handling the issue of laterality by means of a quantitative, meta-analytic approach.

## Figures and Tables

**Table 1:** The key studies reviewed, divided for the different spatial laterality tasks (i.e., physical and numerical bisection, mirror symmetry, and localization tasks), along with the number of sighted and blind participants involved.

Author	Year	Blind Participants	Control Participants
***Pseudoneglect (line bisection)***			
Bradshaw et al. [13]	1986	10	24
Cattaneo et al. [14]	2018	11	25
Cattaneo, Fantino, Tinti et al. [15]	2011	17	18
Sampaio et al. [16]	1995	20	20
***Pseudoneglect (numerical bisection)***			
Cattaneo, Fantino, Silvanto et al. [17]	2011	18	10
Cattaneo, Fantino, Tinti et al. [18]	2010	17	23
Rinaldi et al. [19]	2015	16	16
***Mirror symmetry***			
Bauer et al. [20]	2015	8	7
Cattaneo, Fantino, Silvanto et al. [21]	2010	16	26
Cattaneo et al. [22]	2013	12	12
***Localization tasks***			
Collignon et al. [23]	2009	6	0
Vercillo et al. [24]	2017	8	8
